# Understanding the Curvature Effect on the Structure and Bonding of MoC_*y*_ Nanoparticles on Carbon Supports

**DOI:** 10.1021/acsami.4c17904

**Published:** 2025-01-17

**Authors:** Wei Cao, Marc Figueras-Valls, Francesc Viñes, Francesc Illas

**Affiliations:** Departament de Ciència de Materials i Química Física & Institut de Química Teòrica i Computacional (IQTCUB), Universitat de Barcelona, c/Martí i Franquès 1-11, Barcelona 08028, Spain

**Keywords:** molybdenum carbide, carbon support, curvature effect, catalyst–support interaction, catalyst design

## Abstract

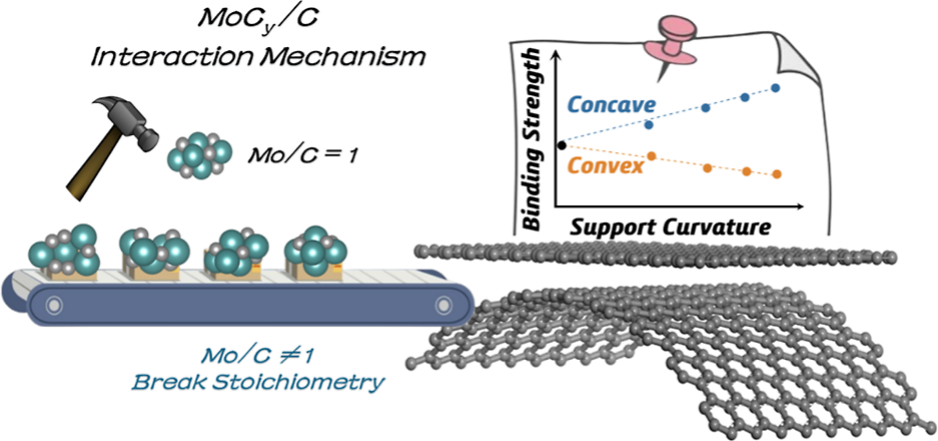

The interaction between molybdenum carbide (MoC_*y*_) nanoparticles and both flat and curved graphene surfaces, serving as models for carbon nanotubes, was investigated by means of density functional theory. A variety of MoC_*y*_ nanoparticles with different sizes and stoichiometries have been used to explore different adsorption sites and modes across models with different curvature degrees. On flat graphene, off-stoichiometric MoC_*y*_ featuring more low-coordinated Mo atoms exhibits stronger interaction and increased electron transfers from the carbide to the carbon substrate. This preferentially occurs through support C and Mo atoms leading to the formation of additional Mo–C bonds. Notably, the MoC_*y*_ adsorption strength increases on concave surfaces and decreases on convex surfaces, showing a strong linear correlation with the surface curvature. This curvature-dependent behavior alters the charge state of the nanoparticles, making them more/less positively charged in concave/convex regions. The present results demonstrate that the interaction strength can be effectively tuned by manipulating the carbide stoichiometry, the substrate curvature, and the local concave/convex environments, providing valuable guidelines for the rational design of MoC_*y*_/C-based catalysts.

## 1 Introduction

The catalytic conversion of carbon dioxide (CO_2_) into other valuable chemicals or fuels is a “*two birds, one stone*” approach, which can help in solving the climate change crisis while alleviating the nowadays energy-deficit situation.^1,2^ The abundant and relatively economic transition metal carbides (TMCs) have been suggested as good alternatives to costly Pt-group noble metals for diverse CO_2_ conversion processes, including the reverse water–gas shift (RWGS) reaction and other CO_2_ hydrogenation processes, where TMCs exhibit comparable or even better activity and selectivity than Pt-group metals.^3,4^ Molybdenum carbides—MoC and Mo_2_C—are broadly studied TMCs and reported as excellent catalysts for a series of important catalytic reactions for CO_2_ utilization, involving, e.g., RWGS,^5^ electrochemical CO_2_ reduction reaction (CO_2_RR),^6^ and methanol synthesis (CH_3_OH), to name a few.^7,8^ These materials are versatile and suitable supports for noble, late transition metals, tuning their electronic structure and, ultimately, their catalytic performance,^9^ while they can act as catalysts themselves.^10^ As supports, cubic δ-MoC, hexagonal α-MoC, and orthorhombic β-Mo_2_C have been used to sustain noble metal cluster catalysts upon, ultimately biasing their catalytic performance;^11,12^ for instance, even for the same metal cluster supported on hexagonal TMCs, the electron transfer direction can vary between them and M-/C-terminated molybdenum carbide surfaces, leading to electron accumulation/depletion on active sites of the supported cluster, which benefits the adsorption of an electron acceptor like CO_2_ or electron donor like hydrogen (H_2_). For instance, δ-MoC (001) with controlled H coverage has been found to efficiently catalyze the hydrogenation of ethylene.^13^ Finally, MoC-specific surfaces have been found to bind and convert CO_2_ to CH_3_OH with a high reactivity.^14^

Unlike extended surfaces, small clusters and nanoparticles (NPs) of MoC with variable stoichiometry, hereafter referred to as MoC_*y*_,^1^ possess distinctive features, such as low-coordinated sites and varying metal/carbon ratios, which can be quite useful for activating key molecules including methane (CH_4_), CO_2_, and H_2_.^15−18^ For instance, MoC_*y*_ NPs supported on a Au (111) surface have been found to be able to activate CH_4_ at room temperature,^15^ while exhibiting a hydrogen sponge effect,^18^ that potentially promotes the CO_2_ hydrogenation to a great extent.^4^ Moreover, the tunable composition and size of MoC_*y*_ NPs not only enable the activation of key molecules but also offer a way to modulate the activity and selectivity. Regarding the size effect, generally larger MoC_*y*_ NPs with lower formation energies per formula unit stand for better thermodynamic stability,^19^ while smaller NPs possess higher surface areas and a larger ratio of low-coordinated active sites. This implies easier structural reconstruction to accommodate molecules upon, therefore enhancing chemical activity, which can be translated into an enhanced catalytic performance.^16^ As far as stoichiometry is concerned, it has been shown that, compared to Mo-rich carbide (MoC_0.6_) NPs, nearly stoichiometric carbide (MoC_1.1_) NPs exhibit an improved stability and selectivity converting CO_2_ to CH_3_OH.^4^ On the other hand, MoC_*y*_ NPs with a Mo/C ratio below 1.08 are predicted to be optimal candidates for distorting C=C bonds, a key step in C_2_H_4_ hydrogenation.^19^

Recently, carbon-based materials such as graphene and carbon nanotubes (CNTs) have been suggested as a support for MoC_*y*_ NPs resulting in a new type of supported catalysts. For instance, Liu et al.^20^ found that MoC_*y*_ NPs/nanoclusters anchored on a core@shell hybrid structure made of nanodiamond@graphene (ND@G) can catalyze the RWGS with better performance than the MoC_*y*_ NPs supported by typical substrates such as activated carbon, γ-Al_2_O_3_, or β-Mo_2_C. Baddour et al.^17^ found that catalysts involving α-MoC_*y*_ NPs on what they termed as an inert carbon support exhibited a 2-fold increase in both the activity and selectivity toward C_2+_ products compared to an unsupported α-MoC_*y*_ analogue. It is noteworthy that the inertness of the carbon support is not fully clear since it critically affects the catalytic performance. Indeed, there is yet no consensus on whether the observed enhanced reactivity is solely due to the disparities between NPs and bulk materials or whether interactions between the carbon support and MoC_*y*_ NPs can play a crucial role. Therefore, investigating the interaction between the carbon support and MoC_*y*_ NPs, further considering the impact of the surface local environment on catalytic performance, is essential to provide answers to the above questions.

From the possible carbon-based materials that can be used as a support for MoC_*y*_ NPs, graphene has undeniably emerged as a prominent candidate, broadly used in catalysis, materials science, and energy-related applications.^21,22^ However, it is worth noting that, due to its sp^2^ hybridization conferring a high degree of aromaticity, pristine flat graphene (FG) has a quite reduced chemical activity. Various methods such as doping, functionalization, and/or strain are often employed to endow a higher catalytic activity to pristine FG.^23−26^ Among the modulations of carbon surfaces, curvature emerges as an important feature, as well. On the one hand, Xu et al.^27^ pointed out that it is challenging for a monolayer of graphene to remain perfectly flat due to thermal fluctuations and practically unavoidable local strain. On the other hand, Sun et al.^28^ found that nanoripples accompanied by the presence of curvature often lead to superior catalytic performance of graphene-based catalysts, which brings the crucial topic of curvature to the forefront of research.^29−31^ For example, Pan et al.^32^ found that for pristine graphene, hydrogen evolution reaction performance increases more than 50% with the presence of curvature, and for B-/N-/metal-doped graphene, the best catalytic ability occurs under certain curvature conditions but not on flat ones. In fact, the chemical activity of curved graphene (CG) is higher than that of the flat one. This is attributed to the presence of the higher pyramidalization angles, a geometrical feature that quantifies the extent to which the central atom is pushed out of this plane due to the strain.^33^ Recently, Kang et al.^34^ employed a hybrid quantum-mechanics/machine-learning (QM/ML) approach to investigate the curvature effect on oxygen reduction reaction catalyzed by Fe–N–C and found the Fe d orbital can be linearly adjusted by curvature which leads to change of the binding strength of key reaction intermediates. In the same line, Liu et al.^35^ found that Co_3_O_4_ NPs confined within CNT nanochannels exhibited superior catalytic performance in the removal of contaminants from water than that of NPs situated on the outer surface. To conclude, the previous studies described above have demonstrated that the curvature of graphene can significantly influence both its own catalytic properties and those of the supported catalysts. However, hitherto, there has been no systematic theoretical investigation on how graphene curvature and the resultant concave and convex surfaces affect its interaction with supported NPs. This missing piece of information is indeed tackled here by focusing on the case of MoC_*y*_ NPs supported on a series of flat and curved carbon surface models.

To this end, we systematically investigated the interaction of MoC_*y*_ NPs with different carbon support models using density functional theory (DFT)-based calculations. By using a family of MoC_*y*_ NPs with different size and composition, carbon substrates with different curvatures, and concomitant convex/concave local environments, one can determine the effect of the surface curvature on the adsorption energy and electron transfer between the interacting systems, with the manipulation of the MoC_*y*_ stoichiometry and substrate curvature being parameters to adjust the interaction. Specifically, it is found that metal-rich MoC_*y*_ NPs with low-coordinated Mo atom sites strongly bind with the carbon support, and the adsorption strength of MoC_*y*_ NPs linearly increases with increasing curvature in concave regions, while it decreases in convex regions, being practical ways to regulate the MoC_*y*_ binding strength on curved carbon supports, which ultimately could affect the overall MoC_*y*_ chemical and catalytic reactivity.

## 2 Modeling Curved Carbon Surfaces

**Figure 1:**
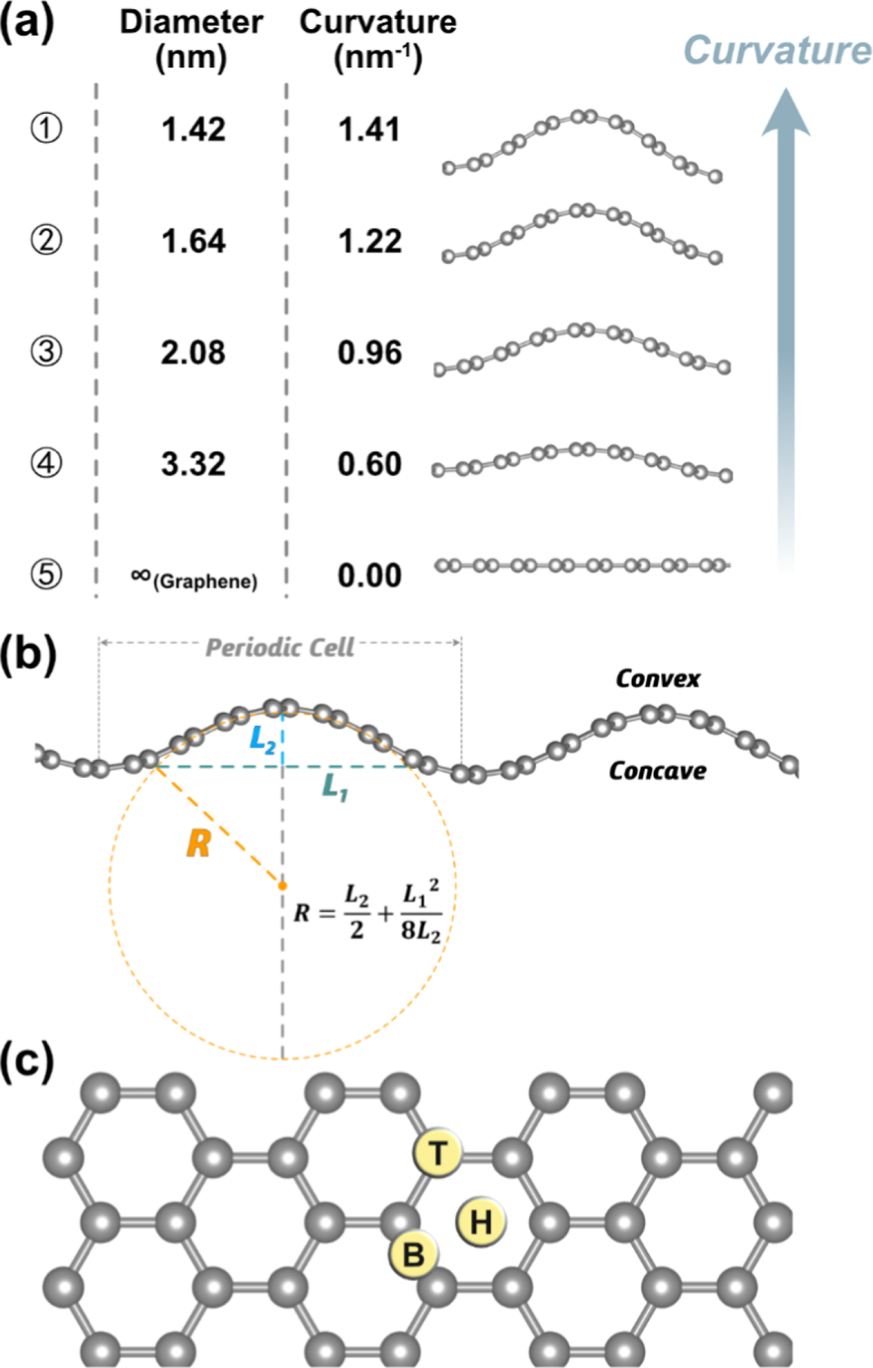
(a) Side views of CG structures and corresponding diameters and curvatures. (b) Scheme indicating how diameters of the CNTs from CG are estimated. Additionally, the convex and concave local regions, as well as the range of the periodic cell, are labeled. (c) Top views of high-symmetry sites, where T, B, and H represent top, bridge, and hollow sites, respectively. C atoms are shown as gray spheres.

Due to the potentially significant impact of curvature on the catalytic potential of graphene-based carbon materials, some theoretical simulation studies have been reported in recent years, aiming to explain and predict its influence, although mainly in electrocatalytic systems,^31−33,36^ and yet with quite limited models. While the carbon-based substrates used in experiments include FG and CNTs with diameters ranging from ∼0.5 nm in single-walled CNT (SWCNT) to ∼10 nm in multiwalled CNTs (MWCNT), theoretical studies focus mainly either on FG^37−39^ or on single-walled CNTs with a diameter of just around 1 nm.^40−42^ This is not surprising since DFT calculations for CNTs models with a realistic diameter of several nanometers are computationally too demanding, up to the point of being unfeasible nowadays. There are two compromise models involving either using CG to simulate a segment of a CNT arc in periodic DFT calculations^30,32^ or relying on quantum-mechanics/molecular-mechanics (QM/MM) or QM/ML methods, where only specific surface sites are calculated using DFT, while other parts use lower-precision but more affordable computational methods like empirical force fields^43^ or ML interatomic potentials.^33^ In the present work, we chose the first option, with periodic models for FG and SWCNTs with diameters in the 1.4–3.3 nm range, encompassing the typical diameters of SWCNTs^44^ and MWCNTs,^45^ respectively, as shown in Figure 1.

In the first step, the flat armchair graphene is modeled by a *c*(8 × 6) supercell containing 96 C atoms, with dimensions of 17.1 Å in length (*a*), 14.8 Å width (*b*), and with a vacuum region of 20 Å in the direction perpendicular to the graphene sheet, to avoid the interaction between the periodically repeated graphene layers. To mimic the SWCNT surfaces of diameter in the above-mentioned range, the unit cell *a* lattice parameter was compressed, leading to an energy that increases uniformly with the compression, as shown in Figure S1a of the Supporting Information, which is consistent with the conclusions reported by Pan et al.^31^ To quantify the curvature of the resulting model, one can rely on the compression percentage, ε, defined as ε = (*a*–*a*_c_)/*a*, where *a*_c_ and *a* stand for the length of the graphene supercell with and without compression. Note, however, that for the same ε, the curvature depends on the initial *a* lattice parameter, which may lead to an inconsistent measure of the curved space. To avoid this flaw, we define the curvature, κ, from its arc length considered as a part of a perfect CNT, see Figure S2, with a geometrical analysis used to estimate the diameter of the corresponding CNT and thereby derive the curvature (reciprocal of the radius, measured in nm^–1^). This definition is consistent with the method used by She et al.^30^ Increasing κ is accompanied by a rise in the total energy (*E*), as in the right panel of Figure S1a. Since the curvature is the reciprocal of the radius, a uniformly smaller radius results in a more pronounced change in curvature under stronger compression. Consequently, the *E* ∼ κ relationship fits a quadratic function more closely than a linear regression. Further details on model construction are shown in Section S1 and Figure S3. The model is validated, e.g., comparing explicit CNT models of a diameter of 1.65 nm with a CG model with an approximated diameter of 1.65 nm, see Figure S4. The adsorption of the Mo_6_C_6_ NP model in the concave/convex regions leads to essentially the same adsorption conformation (cf. Figure S4), with equivalent adsorption strengths and local deformations; see values in Table S1.

Note that even when focusing on the *a* direction, different compression directions of the cell (*a* vs *b*) result in distinct crystallographic edge orientation (armchair vs zigzag).^46^ Taking the adsorption of Mo_6_C_6_ NP on CG with the same curvature as an example, as shown in Figure S1, the influence of the compression direction on the adsorption energy of Mo_6_C_6_ NP on the carbon support is almost negligible. In fact, even if compression along zigzag is somewhat more energetically costly, as found in previous reports,^47^ the impact on the Mo_6_C_6_ NP adsorption is minimal, with variations in energy below 0.02 eV, and very similar minima structures. Thus, in the following, only armchair compressions—along the *a* vector—are contemplated, to ease the discussion.

## 3 Computational Details

**Figure 2:**
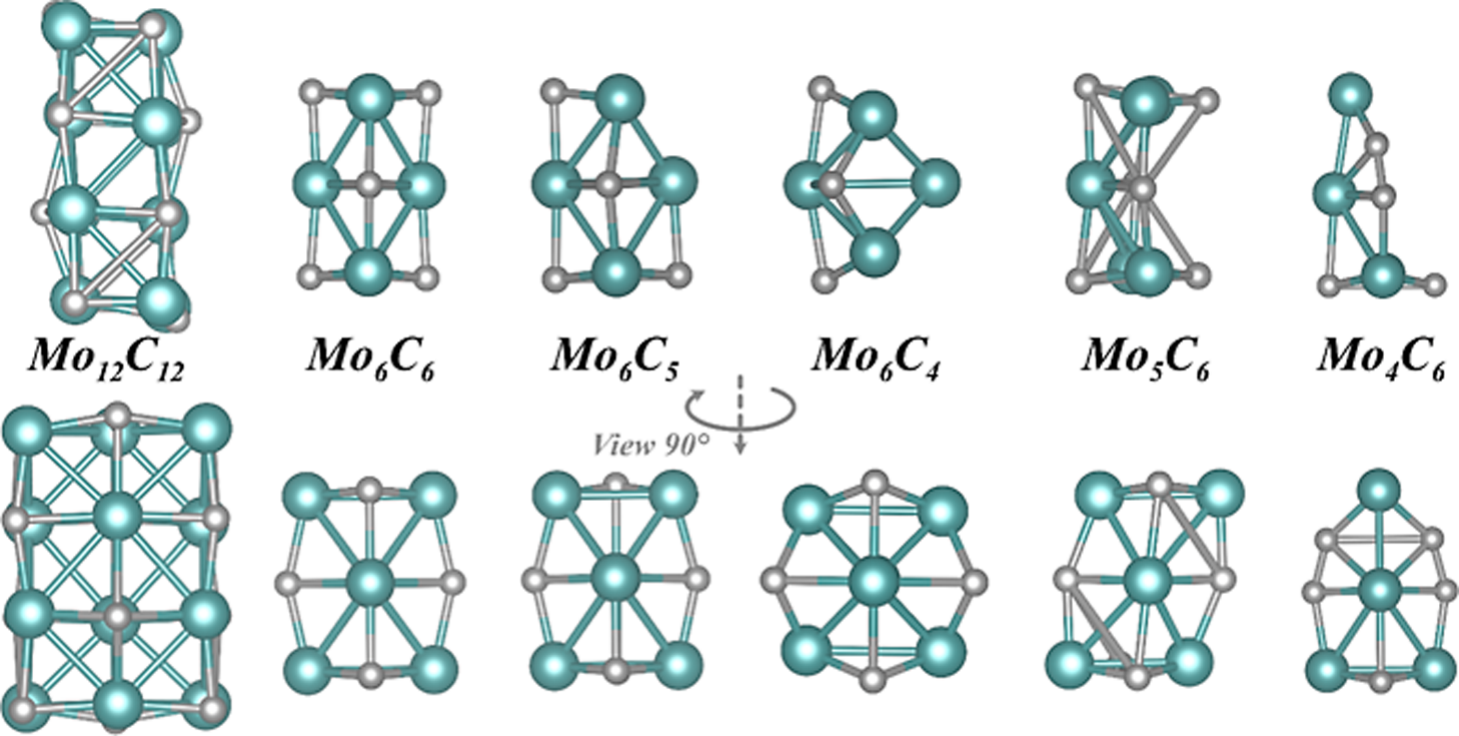
Atomic structures of the most stable MoC_*y*_ NPs in vacuum. Cyan spheres denote Mo atoms, while the rest of the color coding is as in Figure 1.

To study the interaction of MoC_*y*_ NPs on the flat and curved carbon surfaces as described above, a series of calculations were carried out in the framework of DFT. The MoC_*y*_ systems considered in this study contain up to 24 atoms, and the Mo/C ratio varies from 0.67 to 1.50, with the atomic structures shown in Figure 2. The set of NPs here comprises stoichiometric ones like Mo_6_C_6_ and Mo_12_C_12_, Mo-rich ones like Mo_6_C_5_ and Mo_6_C_4_, and C-rich ones like Mo_5_C_6_ and Mo_4_C_6_. The structures of these MoC_*y*_ clusters reported and confirmed as stable isomers by Jiménez-Orozco et al.^19^ were used as starting points. Note that even if such studied MoC_*y*_ isomers size is below 1 nm, we would refer to them in the following as NPs.^48^

All calculations were conducted employing the Vienna ab initio simulation package (VASP) code,^49^ utilizing the Perdew–Burke–Ernzerhof (PBE) exchange–correlation functional,^50^ which has been identified as appropriate for describing Mo-based carbides.^51^ The contribution of dispersion interactions to the total energy was accounted for through Grimme’s D3 method.^52^ The Kohn–Sham equations were iteratively solved by expanding the valence electron density in a plane-wave basis set with a cutoff of 415 eV, enough to acquire converged results with chemical accuracy. Additionally, the effect of core electrons on the valence electron density was taken into account using the projected augmented wave method,^53^ as implemented in VASP by Kresse and Joubert.^54^ The effect of spin polarization has been systematically investigated in selected systems and was found to be negligible in all cases, consistent with previous works,^4,18,19^ and so disregarded in the following. Convergence thresholds for the convergence of total energy and atomic forces acting on atoms were set to 10^–5^ eV and 0.01 eV·Å^–1^, respectively. For chemical bonding analysis, the Bader charges are calculated using the code by Henkelman et al.^55^ In all cases, sufficiently large cells were used, as described in detail in the next section. These ensured that the interaction between supported NPs in periodically repeated images is negligible, below 0.01 eV per MoC unit; see Table S2. For the isolated MoC_*y*_ NPs, calculations were carried out at the **Γ** point, whereas for MoC_*y*_ NPs supported on carbon substrates, a 3 × 3 × 1 Monkhorst–Pack grid of special **k**-points was used.

The stability of the resulting MoC_*y*_/carbon composites has been analyzed by a series of energetic variables: the adsorption energy *E*_ads_, when MoC_*y*_ NPs are supported on carbon substrates, either FG or CG; the deformation energy *E*_def_, which describes the energy required to deform either the support or the MoC_*y*_ NPs from its isolated optimized atomic structure to their geometries on the composite material; and the attachment energy, *E*_att_, accounting for the interaction energy between the already deformed units. The above quantities are thus defined as
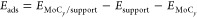
1
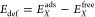
2
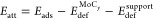
3where  corresponds to the total energy of the composite; *E*_support_ is the total energy of FG or CG support; *E*_*MoCy*_ is the total energy of the corresponding NP in the gas phase; *E*_*X*_^ads^ and *E*_*X*_^free^ represent the total energy of an isolated *X* entity (NP or support) with the exact same geometry as adsorbed in composite and the total energy of the optimized entity in the gas phase, respectively; and  and *E*_def_^support^ indicate the deformation energy of the NP and support, respectively. Finally, note also that, by construction
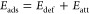
4Within the definitions above, the formation of MoC_*y*_ composites on any of the explored substrates is favorable when *E*_ads_ < 0, as the adsorption of the NPs would be an exothermic process. Similarly, negative values of *E*_att_ indicate a favorable interaction, while *E*_def_ is positive and can be viewed as the energy cost to deform the NP or the support to reach the energy minimum of the composite.

## 4 Results and Discussion

The atomic structures of a range of MoC_*y*_ NPs with varying sizes and metal/carbon ratios will be discussed first. For consistency, the structures reported in previous works^4,19,56^ were reoptimized in a cubic vacuum cell with a side length of 20 Å, and the resulting, almost identical, geometries were subsequently loaded onto the FG and CG models. It is worth pointing out that the geometry optimization for Mo_6_C_4_ delivered a negative *E*_def_, implying the existence of a lower-lying isomer with C_4v_ symmetry, see Figure S5, at variance with the C_2v_ isomer as previously reported.^1,15,57^

### 4.1 MoC_*y*_/FG Composites

**Figure 3:**
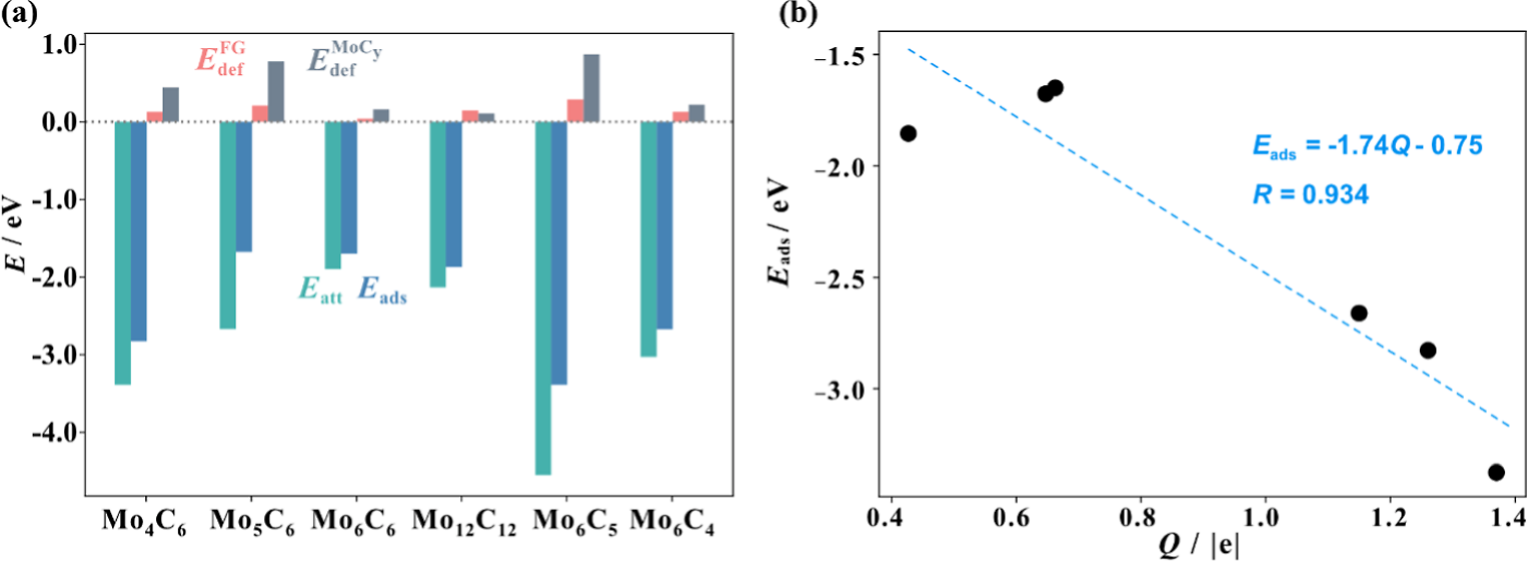
(a) Energy variables of MoC_*y*_ NPs adsorbed on FG on their most stable configurations; *E*_ads_, *E*_att_, *E*_def_^*FG*^, and  and (b) linear relationship between *E*_ads_ and the MoC_*y*_ Bader charges, *Q*, on FG.

As a reference, the interaction of MoC_*y*_ with the FG model was first analyzed, for which several sites have been considered, see Figure 1c. To identify the thermodynamically most stable adsorption configurations of each NP on FG, all representative adsorption configurations, conformations, and orientations were systematically tested. The most stable adsorbed configurations are thus shown in Figure S6, along with the less stable ones and their corresponding relative energies, Δ*E*. The calculated values of *E*_ads_, *E*_def_, and *E*_att_ are summarized in Table S3 and plotted in Figure 3a. As depicted, stoichiometric Mo_6_C_6_ and Mo_12_C_12_ exhibit a similar adsorption strength on FG, while nonstoichiometric NPs generally exhibit stronger interactions, as indicated by larger *E*_ads_, compensating more positive *E*_def_ (for both FG and NPs) and with negative *E*_att_ values, where Mo_6_C_5_ shows the strongest adsorption.

**Figure 4:**
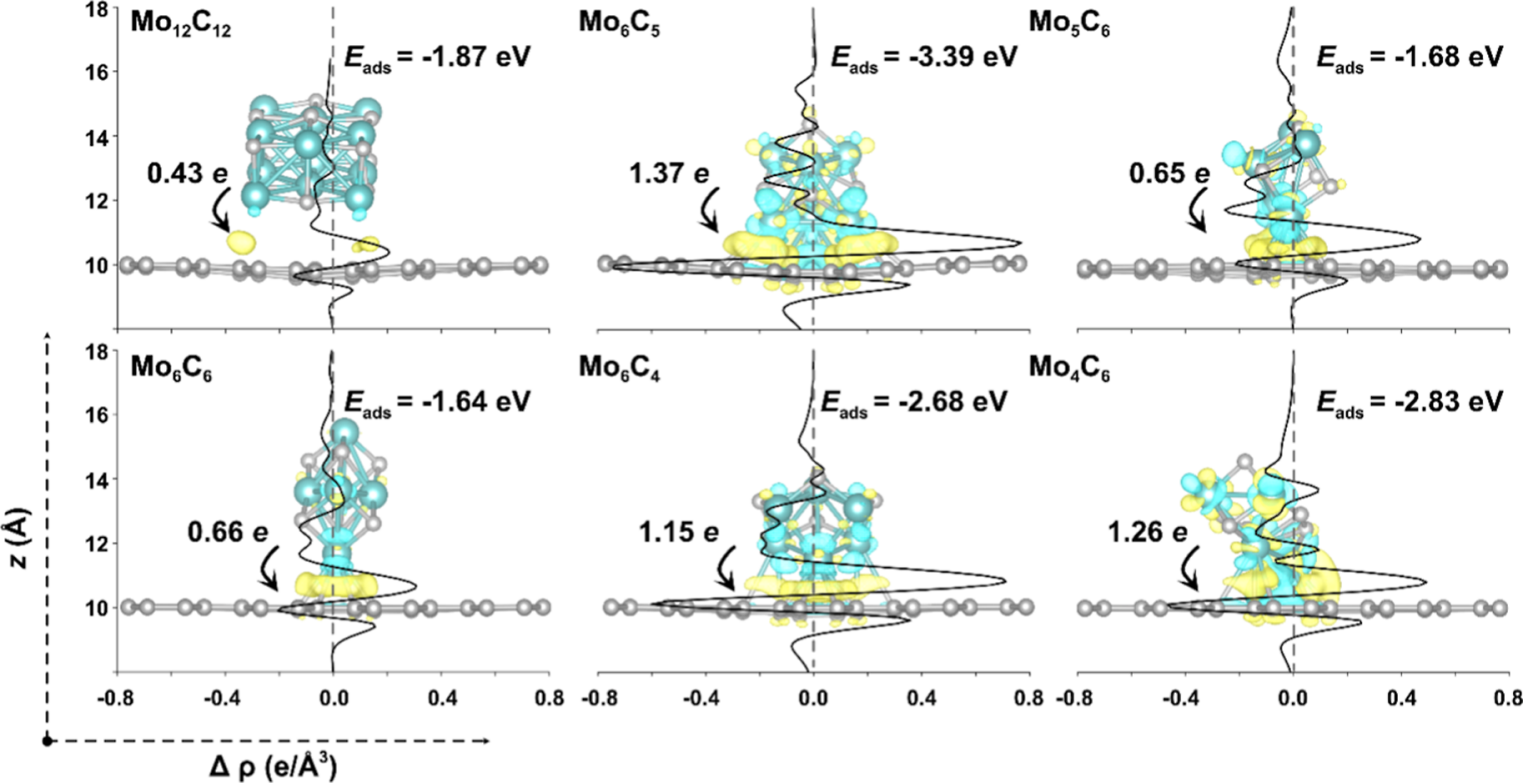
Side views of the atomic structures of the most stable adsorption configurations of MoC_*y*_ NPs on FG, along with CDD plots. Electron depletion/accumulation isosurfaces are depicted in blue/yellow at isovalues of ±0.03 e Å^–3^. The black arrows and numbers indicate the direction and magnitude of the total charge transfer, while black lines are plane-averaged CDD, Δρ. Color-code as in Figure 2.

Overall, regardless of the stoichiometry, MoC_*y*_ NPs bind stronger on FG through the formation of Mo–C bonds, in such a way that the larger the number of Mo–C bonds, the stronger the *E*_ads_ is, as a way of saturating low-coordinated Mo atoms. Figure 4 displays side views for the strongest adsorption structure of each NP on FG, along with the corresponding charge density difference (CDD) plots and plane-averaged CDD along the graphene sheet surface’s normal direction. As evident in Figure 4, an electron accumulation is observed at the interface plus a charge transfer from MoC_*y*_ to graphene.

Regarding stoichiometric NPs, the most stable case for Mo_12_C_12_ on FG is not a flat configuration, as reported, e.g., for Mo_12_C_12_ on a Au(111) support,^4^ but rather a tilted configuration, as shown in Figure 4. Here, a Mo-rich cuboid Mo_12_C_12_ NP edge is in contact with FG, with an adsorption energy *E*_ads_ = −1.87 eV and a charge transfer from the Mo_12_C_12_ NP of 0.43 e. The coordination number (CN) of these Mo atoms is just three, making them more prone to interact with FG. It is worth noting that two other adsorption configurations were found, which are reported in Figure S6. One of these configurations essentially isoenergetic with the most stable one described above implies the bonding through a corner Mo atom, while the other implies the interaction with the longer edge. Note also that for edge contacts, there is a sensible deformation of the graphene layer, repelled from the MoC_*y*_ NP, with a *E*_def_^FG^ of approximately 0.15 eV, as presented in Table S3. Conversely, corner interaction leads to an almost negligible graphene deformation energy of 0.05 eV only; see Figure S7a. These differences can be attributed to Pauli repulsion between the C atoms of graphene and those at the MoC_*y*_ edges. The situation regarding Mo_6_C_6_ is analogous to that of Mo_12_C_12_, where two adsorption configurations exhibit comparable energetic stability, see Figure S7b. Both the edge and corner configurations have *E*_ads_ differing by less than 0.1 eV, and so in principle competitive, with charge transfers from MoC_*y*_ toward FG of 0.66 e for both cases. Compared to the edge configuration, the corner configuration again exhibits smaller repulsion between FG and MoC_*y*_ C atoms, resulting in smaller deformation of the FG, where the *E*_def_ of the FG is 0.2 eV lower than in the edge case.

When it comes to nonstoichiometric MoC_*y*_ NPs, in Mo_6_C_5_, the removal of one C atom compared to Mo_6_C_6_ disrupts the rock-like structure, exposing more low-coordinated Mo sites, which would therefore strengthen the adsorption, as indeed observed with an *E*_ads_ of −3.39 eV for Mo_6_C_5_, accompanied by a stronger electron transfer, now of 1.37 e, plus more evident electron accumulation/depletion isosurfaces at the Mo_6_C_5_/FG interface, see Figure 4. More intuitively, the plane-averaged CDD, Δ*ρ*, along the *z* direction fluctuates more severely compared to the supported stoichiometric counterparts, which implies stronger chemical bonding between Mo_6_C_5_ and FG. The stronger adsorption of Mo_6_C_5_ compared to Mo_6_C_6_ underscores the significant role played by the Mo/C atomic ratio, where the presence of more low-coordinated Mo atomic sites enhances the adsorption strength. To further validate this hypothesis, note that the adsorption of Mo_6_C_5_ through a single corner Mo site has an *E*_ads_ of −1.80 eV, very similar to the equivalent situation on Mo_6_C_6_ with an *E*_ads_ of −1.70 eV, see Figure S6. In essence, the strengthening effect on Mo-rich MoC_*y*_ NPs is primarily attributed to atomic rearrangements, where closely spaced, low-coordinated Mo atoms interact with the carbon support rather than a significant change in the overall electronic structure.

Following the analysis, the Mo_6_C_4_ NP also exhibits stronger adsorption on FG compared to Mo_6_C_6_, since again removing two C atoms exposes more low-coordinated Mo sites, in line with the above discussion. However, the adsorption strength of Mo_6_C_4_ falls between that of Mo_6_C_5_ and Mo_6_C_6_. In both Mo_6_C_5_ and Mo_6_C_4_, a Mo atom adsorbs at a hollow site on FG, but due to the high geometric symmetry of Mo_6_C_4_, neighboring Mo atoms compete for adsorption. This competition prevents Mo_6_C_4_ from approaching the graphene surface as closely as in the case of Mo_6_C_5_, leading to a weaker interaction and adsorption strength, as shown in Figure S8. It is also worth mentioning that, as shown in Figure 3, *E*_def_ for the Mo_6_C_4_ NPs on FG (0.22 eV) is much smaller than that of Mo_6_C_5_ (0.87 eV). To gain a deeper understanding of , the RMSD with respect to the gas phase structure was calculated for all MoC_*y*_ NPs atoms when adsorbed on FG, providing a quantitative measure of the cluster deformation extent, see detailed values in Table S3. Two types of deformations were observed: small deformations with an RMSD below 0.10 Å and *E*_def_ values below 0.5 eV and significant deformations in Mo_6_C_5_ and Mo_5_C_6_, with RMSD above 1.8 Å and *E*_def_ exceeding 0.75 eV. However, the limited data points make it difficult to capture one or more trends adjustable to a function.

Regarding C-rich MoC_*y*_ NPs, the morphology of Mo_5_C_6_ NP is similar to that of Mo_6_C_6_, but having one less Mo atom breaks the rock-salt-like structure and results in a relatively low-symmetry structure. The most stable adsorption configuration of Mo_5_C_6_ NPs on FG is also like that of Mo_6_C_6_, contacting FG through the NP edge. However, it is worth noting that due to the relatively loose internal structure of the Mo_5_C_6_ NP, its deformation upon contact FG is evidenced by the reduction in the Mo–C–Mo edge angle from 204.5° to 176.0°, as shown in Figure S9. The central C atom of the edge relaxes toward the interior to reduce repulsion with the graphene sheet, thereby enhancing the interaction between the corner Mo atoms and FG. Consequently, compared to Mo_6_C_6_, both the NP and FG exhibit greater deformation energies during the adsorption of Mo_5_C_6_, leading to a stronger *E*_att_, while *E*_ads_ and electron transfer values remain similar, as shown in Figures 3a and 4. In the case of Mo_4_C_6_, the situation significantly differs; first, Mo_4_C_6_ has the lowest symmetry among the studied MoC_*y*_ NPs, with a C_1_ point group, see Figure 2. This results in the presence of a highly unsaturated Mo atom with a CN of 2, which becomes strongly attached to the FG, with an *E*_ads_ of −2.83 eV and an electron transfer of 1.26 e to the graphene sheet, while the strong interaction between them is confirmed by the sharp peaks in Δ*ρ* along the *z* direction, similar to those observed for Mo_6_C_5_ and Mo_6_C_4_ NPs, as shown in Figure 4. Considering the interactions of all NPs adsorbed on FG, a linear correlation between *E*_ads_ and the degree of electron transfer is observed, as shown in Figure 3b, indicating that a stronger adsorption is linked to a larger electron transfer, accompanied by more pronounced electron density polarization at the interface, see CDDs in Figure 4.

In short, the interaction of MoC_*y*_ NPs on FG revealed that (i) regardless of the stoichiometry, low-coordinated Mo atoms in MoC_*y*_ serve as anchoring points on FG, with adsorption configurations that form more Mo–C bonds resulting in stronger interactions; (ii) the electron transfer consistently occurs from MoC_*y*_ NPs to the graphene sheet, with stronger adsorption leading to greater electron transfer, exhibiting a strong linear correlation; and (iii) by deviating from stoichiometry and disrupting the salt-like structure, off-stoichiometric MoC_*y*_ NPs tend to expose more low-coordinated Mo atoms, resulting in varying degrees of increase in the absolute values of *E*_att_, *E*_ads_, and electron transfer, thereby offering a means to regulate the interaction strength.

### 4.2 MoC_*y*_/CG Composites

Following the above analysis on MoC_*y*_ NPs supported on FG, the interaction of stoichiometric Mo_12_C_12_ and Mo_6_C_6_ NPs on CG models is addressed, with the ultimate goal of securing the curvature effect on them. Unlike the FG case, where edge and corner adsorption configurations showed similar adsorption strengths and comparable electron transfer values as shown in Figure S7, the differences are more pronounced on CG models. For instance, while the edge middle C atom of MoC_*y*_ faces strong repulsion from the FG support, the increased distance in the concave CG regions mitigates this repulsion. This allows Mo_12_C_12_ and Mo_6_C_6_ NPs to naturally transition to a swing configuration where both Mo corner atoms interact with the CG C atoms, especially on concave CG models, see Figure S10.

**Figure 5:**
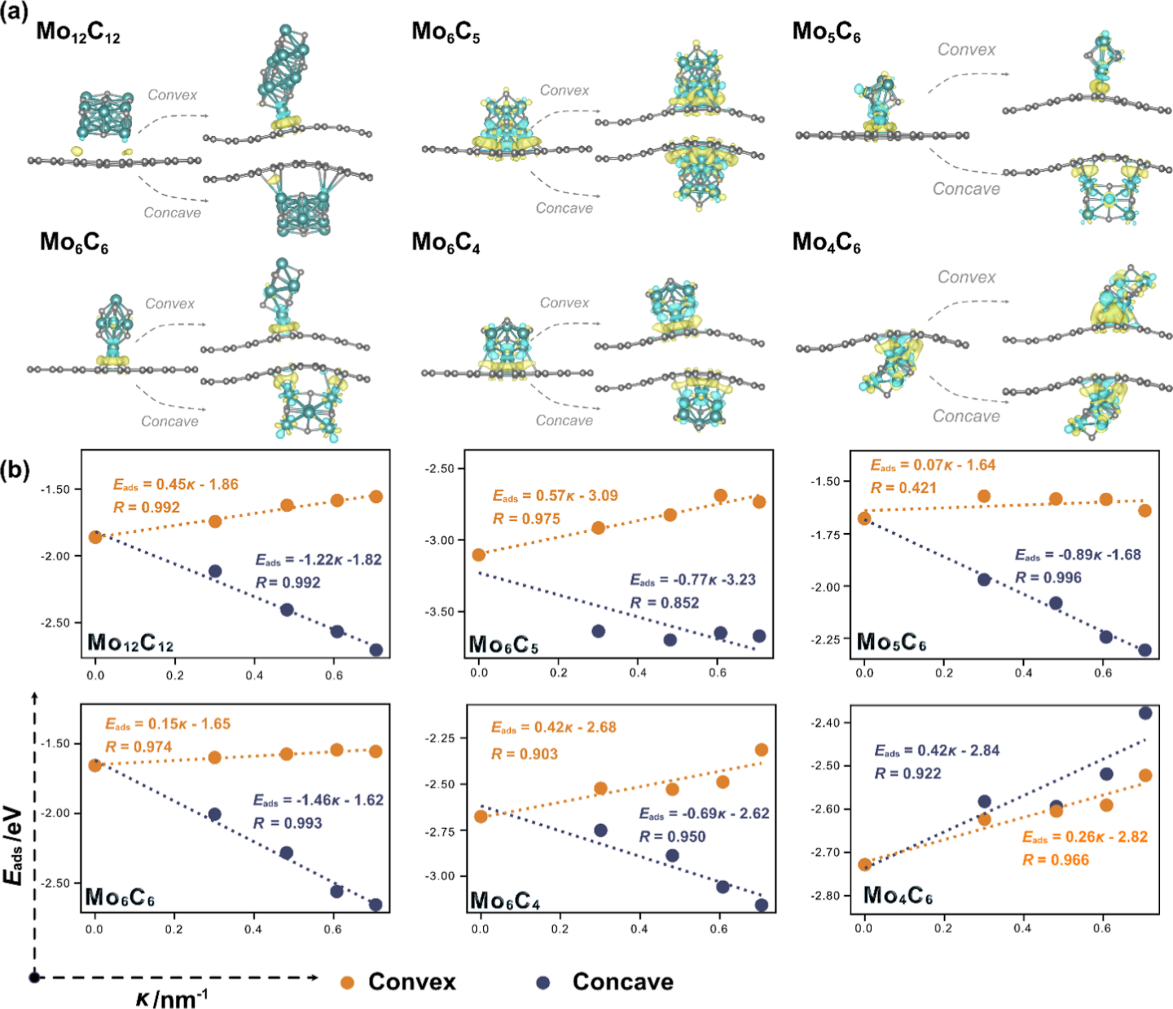
(a) Representative atomic structures of the most stable adsorption of MoC_*y*_ NPs on CG, along with the CDD plots, and whole configurations are depicted in Figure S10. The electron depletion/accumulation is depicted by blue/yellow isosurfaces at ±0.03 |e| Å^–3^. Gray and cyan spheres denote C and Mo atoms, respectively. (b) Adsorption energies of various MoC_*y*_ NPs on concave/convex surfaces of CGs with different curvatures, along with their linear relationships between adsorption energy and curvature.

Additionally, the MoC_*y*_ corner Mo atoms achieve higher coordination with the concave curved carbon atoms, see Figure S10, which is accompanied by a more pronounced electron transfer from MoC_*y*_ to CG, indicative of a stronger bond. This is in line with reports confirming that on CNTs, the electron density on the concave side is found to be lower than on the convex side,^58,59^ with the electron transfer occurring from the NPs to the support. As expected, edge adsorption is generally favored in concave CG, while corner adsorption is preferred on convex CG. As the curvature of the concave region increases, the MoC_*y*_ NPs exhibit larger *E*_ads_ (cf. Figure 5) and get increasingly positively charged (cf. Figure S11). In fact, strong linear relationships are observed between *E*_ads_ of stoichiometric MoC_*y*_ NPs and the degree of curvature, with *R* values of ∼0.992 for Mo_12_C_12_ and ∼0.993 for Mo_6_C_6_, as shown in Figure 5. Based on this, the concave curvature of CNTs is presented as a method for tuning the adsorption strength, charge, and site availability of stoichiometric MoC_*y*_ NPs, factors that may have implications for catalysis, aligning with previous findings^30,31^ that underscore that a specific curvature can lead to an optimal catalytic performance.

Shifting to the convex regions, as introduced above, significant differences are observed. The relative stability of the adsorbed configurations shifts, with the corner adsorption showing stronger bonding and greater thermodynamic stability compared to the edge adsorption. This is due to the aforementioned electron density accumulation on convex surfaces and structural factors that prevent interaction through the MoC_*y*_ edge. In convex regions, the distance between the MoC_*y*_ corner Mo atoms and the support increases, while the distance between the MoC_*y*_ edge C atoms and the support decreases, both contributing to a weaker edge interaction. In contrast, the corner adsorption configuration is less affected by the protrusion of carbon atoms in convex regions. Yet, compared to the adsorption on FG, the adsorption energies slightly decrease with curvature, accompanied by reduced charge transfer, see Figures 5 and S11. As the outside–inside electron density difference of CNTs and CG becomes larger with increasing curvature,^57^ the effect on the convex side may inhibit the electron transfer from MoC_*y*_ NPs to CG, ultimately weakening the interaction. This is reflected in the strong linear relationship observed between the weaker *E*_ads_ of MoC_*y*_ NPs on convex sites and the degree of curvature, with *R* values of ∼0.992 for Mo_12_C_12_ and ∼0.974 for Mo_6_C_6_, see Figure 5.

For Mo-rich Mo_6_C_5_ and Mo_6_C_4_ NPs, the bonding features on concave and convex CG surfaces are similar to those of the stoichiometric NPs, with adsorption strength following the trend: concave > flat > convex. In concave regions, the adsorption strength increases with greater curvature, while in convex regions, the opposite trend is observed; see Figure 5. Specifically, the atomic structure of adsorbed Mo_6_C_5_ includes three low-coordinated Mo bonding atoms, determining the same adsorption on CG as on FG, both in concave and convex regions. The electron transfer direction from MoC_*y*_ to CG remains consistent, with the degree of electron transfer increasing in line with *E*_ads_ and vice versa. Consequently, the MoC_*y*_ NP becomes more positively charged in the concave regions with increasing curvature, while being less positive in the convex regions, see Figure S11. For Mo_6_C_4_, its high structural symmetry (C_4v_) and conical shape create a prominent low-coordinated Mo site at the apex, which serves as an anchoring point for interaction with CG. Similar to Mo_6_C_5_, its adsorption configuration on both concave and convex sides of CG is the same as on FG. The trends in *E*_*ads*_ and electron transfer for Mo_6_C_4_ are also following those of just discussed Mo_6_C_5_.

Interestingly, compared to Mo-rich and stoichiometric NPs, C-rich MoC_*y*_ molecules interact with CG in a different fashion. Let us consider Mo_5_C_6_ first which has a morphology like that of Mo_6_C_6_. Here, the same swing configuration is found on concave CG. As the curvature increases, the *E*_ads_ strengthens, and MoC_*y*_ Bader charges become more positive, with both quantities showing a strong linear correlation, as seen in Figure 5. Still, the absence of one Mo atom in Mo_5_C_6_ makes its structure internally loose. As observed on the FG, edge adsorption causes the central C atom to relax inward, strengthening interactions. Notably, this structural flexibility of Mo_5_C_6_ counteracts curvature-induced weakening interaction in convex regions, making it less sensitive to the model, as indicated by the small positive slope and low linear regression coefficient in the *E*_ads_ vs *κ* plot, see Figure 5. Unlike Mo_6_C_6_, which favors corner adsorption in convex regions, Mo_5_C_6_ prefers edge adsorption. The loose internal structure allows the central C atom to relax inward, similar to its interaction with FG, supported by the higher *E*_def_ of Mo_5_C_6_ in the convex regions, compared to the stoichiometric NPs, as shown in Table S4. The inward relaxation reduces C–C repulsion and enhances the Mo–carbon substrate interaction. Here, two opposing factors influence the adsorption of Mo_5_C_6_: On the one hand, an increased curvature leads to higher electron density in the convex regions,^57^ weakening Mo_5_C_6_ adsorption with reduced charge transfer, while, on the other hand, the intrinsic structural flexibility of Mo_5_C_6_ enhances its adsorption. These competing effects result in weak correlations between *E*_ads_ and curvature in the convex regions.

Finally, regarding the Mo_4_C_6_ NP with C_1_ symmetry, the existence of low-coordinated Mo atoms and distinct C=C dimers result in specific interactions with the CG. Whether in concave or on convex regions, the adsorption configuration consistently positions the lowest-coordinated Mo atom directly at a hollow site, with the adjacent Mo atom at a top or bridge site, as shown in Figure S10. This specific coordination causes the adsorption strength of Mo_4_C_6_ on both the inner and outer sides of CGs to decrease as curvature increases, deviating from the general trend observed in other MoC_*y*_ NPs. Compared to Mo–C bonding, the π–π repulsion between the Mo_4_C_6_ C=C dimers and the carbon support, as shown in Figure S12, has a significant impact on the adsorption strength. As shown in Figure 5, the steeper slope of the *E*_ads_ vs *κ* plot in concave regions indicates that these regions are less favorable for adsorption than the convex ones.

**Figure 6:**
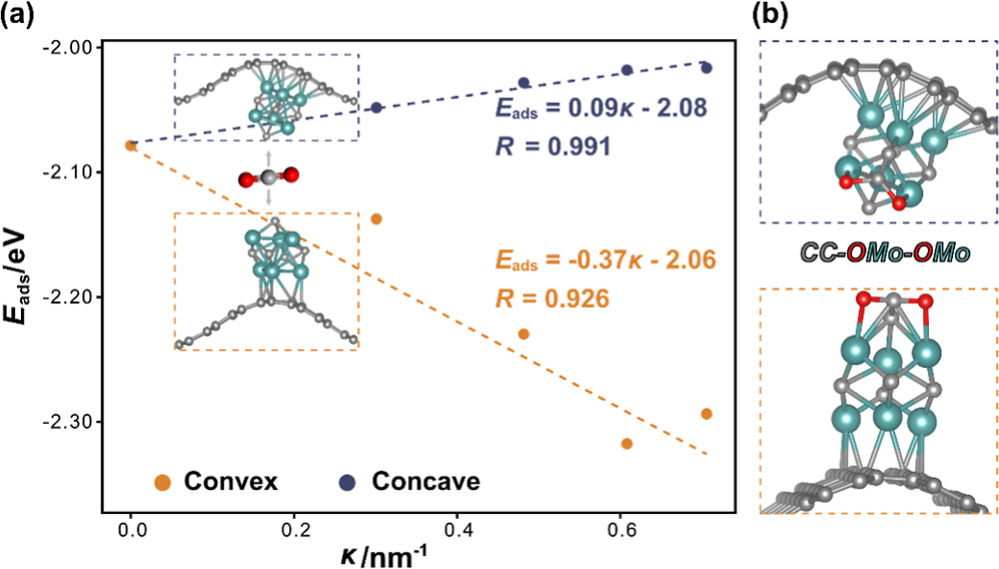
(a) Adsorption energies, *E*_ads_, of CO_2_ on Mo_6_C_5_/CG catalysts with varying support curvatures, along with the linear relationship between adsorption energy and curvature. (b) Atomic structures of the most stable CO_2_ adsorption configurations on Mo_6_C_5_/CG. Oxygen atoms are represented by red spheres, while the rest of the color coding is as in Figure 2.

At this point, having demonstrated the tunable properties of MoC_*y*_/CG models with respect to the model curvature, it is worth assessing the possible chemical activity impact. To this end, we selected Mo_6_C_5_/CG catalysts as an exemplary system and evaluated the CO_2_ interaction on concave and convex models as a function of the CG curvature. As shown in Figure 6a, two distinct trends emerge depending on whether the MoC_*y*_ NP is attached on the concave or convex regions. In the case of concave regions, there is a slight variation with curvature of less than 0.1 eV, weakening the CO_2_ adsorption, even if CO_2_ gets chemically attached, with a bent geometry (cf. Figure 6b). In convex regions, the molecule gets activated as well, but the variation of *E*_ads_ is more pronounced, up to almost 0.3 eV stronger with curvature. These trends align with MoC_*y*_ NP atomic charges as shown in Figure S11, in that MoC_*y*_ get more positively charged in concave regions, and vice versa in convex ones, succinctly implying that a charge transfer to the chemisorbed CO_2_ is easier for convex models. Note in passing by that the activated CO_2_ features a typical C–C bond, and two O–Mo bonds, as shown previously on CO_2_ interaction with MoC_*y*_ NPs.^60^ Also similarly, the CO_2_ bond lengths increase by ∼0.14 Å on average, and the molecular angle reduces to 123.6° on average.

## 5 Conclusions

A comprehensive analysis on the interaction between MoC_*y*_ NPs and carbon substrates, as emerging from DFT-based calculations using the PBE-D3 functional, is presented. The explored substrates include FG and the simplified CG models simulating CNTs walls. The results reveal that both MoC_*y*_ stoichiometry and support curvature significantly affect the interaction strength as well as the degree of electron transfer. On FG, the MoC_*y*_ stoichiometry disruption enhances adsorption due to stronger Mo–C bonding involving low-coordinated Mo atoms, leading to increased electron transfer from the carbide NPs to the substrates. On CG, as in CNTs, the adsorption strength of MoC_*y*_ NPs increases on concave surfaces and decreases on convex surfaces, with a robust linear relationship between adsorption energy and support curvature. The curvature-dependent behavior alters the charge state of the NPs, making them more/less positively charged in concave/convex regions and offering a route to tune catalytic properties via support curvature. Using Mo_6_C_5_/CG as a representative case for CO_2_ adsorption, it is demonstrated that NPs in convex regions are more effective at donating electrons to adsorb and activate CO_2_, compared to those in concave regions.

The present findings highlight the potential to tune the chemical activity of supported MoC_*y*_ on graphene-based substrates by manipulating the carbide stoichiometry, support curvature, and local concave/convex interactions. This insight provides arguments for a rational design of MoC_*y*_/C catalysts with improved catalytic performance.

## Supporting Information Available

Details of constructing CG models; figures including energy changes with compressed ratio and curvature, schematics, comparison of two different kinds of simplified models simulating CNTs, comparison of Mo_6_C_6_ adsorption configurations on a CNT model and a CG model, two orthogonal atomic structure views of Mo_6_C_4_, geometric structures, different views of adsorption configurations with CDD plots and energetic variables, morphology comparison, Bader charges, and charge density plots and heat map; and tables including values of calculated RMSD, adsorption energies, total energy, deformation energy, and attachment energy (PDF)
