# Supplementary material for: Understanding the Curvature Effect on the Structure and Bonding of MoCy Nanoparticles on Carbon Supports
Source: ACS Appl Mater Interfaces. 2025 Jan 17;17(4):7098–108. doi: 10.1021/acsami.4c17904 (PMC11788989; doi:10.1021/acsami.4c17904)

# Understanding the Curvature Effect on Structure and Bonding of $MoC_y$ Nanoparticles on Carbon Supports

Wei Cao, Marc Figueras-Valls, Francesc Viñes, Francesc Illas*

*Departament de Ciència de Materials i Química Física & Institut de Química Teòrica i Computacional (IQTCUB), Universitat de Barcelona, c/ Martí i Franquès 1-11, 08028, Barcelona, Spain*

*Corresponding Author: francesc.illas@ub.edu

**Figure S1.** (a) Energy changes of curved graphene of crystallographic edge orientation armchair and zigzag, with compressed ratio, $\varepsilon$, and curvature, $\kappa$, along with regression formulas, including the regression coefficient, $R$. (b) Schematic figure of compression to get forementioned models, and $E_{ads}$ difference of $Mo_6C_6$ nanoparticle (NP) adsorption in concave and convex regions of curved graphene (CG) with same $\kappa$ (0.6 nm$^{-1}$), respectively. Gray and cyan spheres denote C and Mo atoms, respectively.

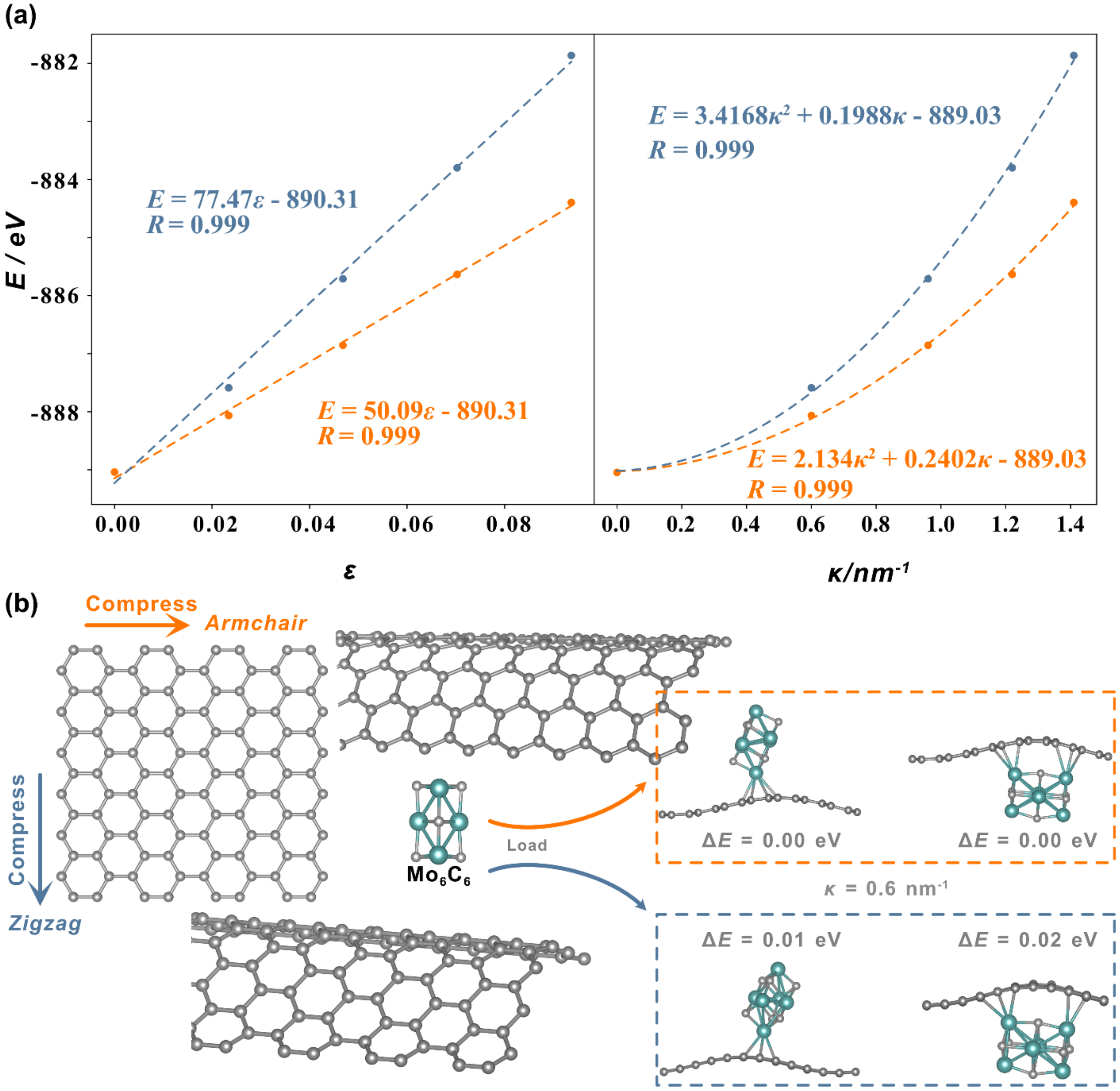

**Figure S2.** Schematic diagram estimating the curvature of curved graphene corresponding to carbon nanotubes using geometric relationships. C atoms are shown as gray spheres.

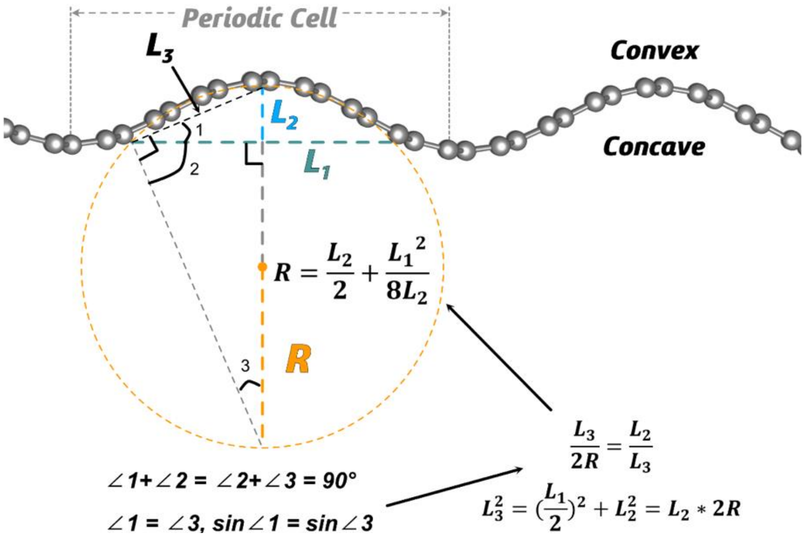

**Section S1: Details to Constructing Curved Graphene (CG) models.**

To simulate Curved Graphene (CG), or to use these models to mimic Carbon Nanotubes (CNT) of certain diameter, one has to notice (*cf.* Figure S3a), that in those carbon atoms are periodically coordinated, and all atoms are allowed to relax during geometry optimizations, at variance with cluster models found in the literature, where a patch of graphene is cut, isolated in vacuum, and capped with H atoms. In such cases, the H atoms are normally fixed to maintain the curvature and avoid the system to relax onto a flat situation. To model continuum curved graphene models, one follows the procedure depicted in Figure S3c. First, departing from a relaxed flat graphene model with sufficiently large vacuum to have the graphene sheet isolated from replicas even when curved, one compresses the unit cell, either along the armchair or zigzag orientations. This is done gradually (*e.g.* in steps of 2% compression), and manually slightly moving a C atom out of the plane to break the symmetry, and avoid unduly structure compressing; in other words, mimicking a perpendicular force. By relaxing the atomic positions at a given compression in a controlled fashion, the rumpled structure of the graphene layer appears, with curvatures dependent on the exerted compression.

**Figure S3.** Comparison of two different kinds of simplified models simulating carbon nanotubes. (a) The CG model as used in the present work. (b) Non-periodic model with edge H atoms to fix the support. (c) The detailed process for constructing the CG models. Color-code as in Figure S1.

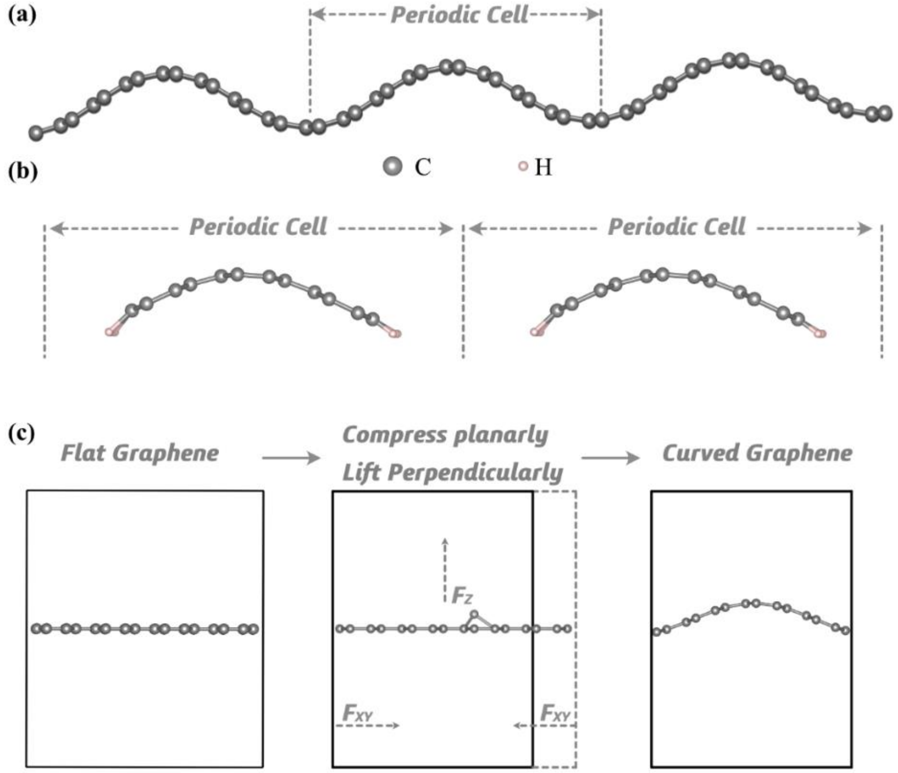

**Figure S4.** Comparison of $Mo_6C_6$ adsorption configurations on a CNT model (upper half) of diameter of 1.65 nm, and a curved graphene model (lower half) of a diameter of 1.64 nm. The configurations on the left represent the initial states, while those on the right are the final states after geometry optimization. Color code as in Figure S1.

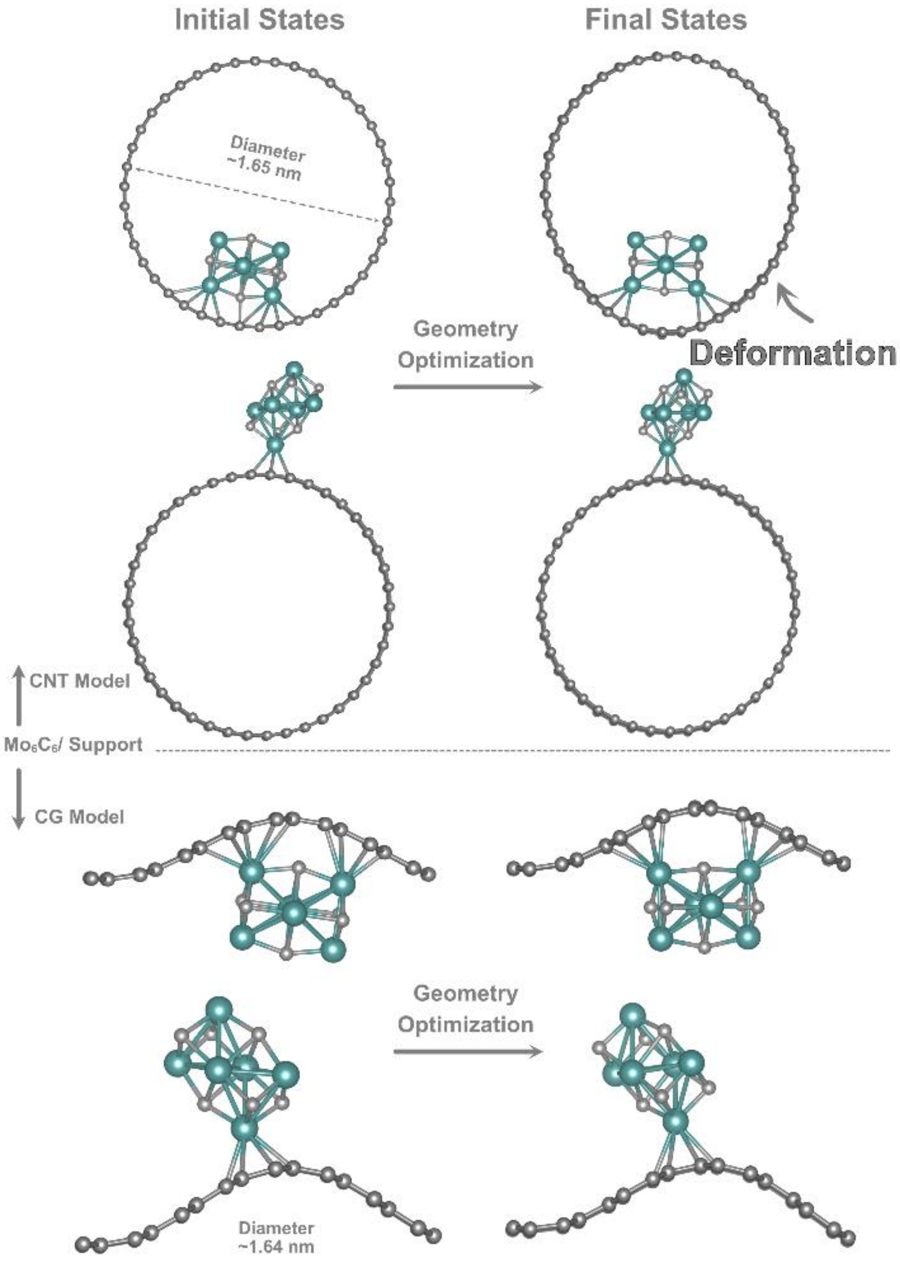

**Table S1.** Calculated Root Mean Square Deviation (RMSD) of the support, and adsorption energies, $E_{ads}$, of $Mo_6C_6$ on the CNT and Curved Graphene (CG) model of Figure S3.

| **$Mo_6C_6$/Support** | **Concave/CNT** | **Concave/CG** | **Convex/CNT** | **Convex/CG** |
|---|---|---|---|---|
| RMSD /Å | 0.35 | 0.17 | 0.02 | 0.01 |
| $E_{ads}$ /eV | -2.59 | -2.56 | -1.54 | -1.54 |

**Table S1.** Total energy, *E*, per MoC unit, of an isolated $Mo_{12}C_{12}$ cluster with different vacuum regions in the periodic unit cell, with lattice length, *a*.

| Structure | ***a*** **(Å)** | ***E*** **(eV)** |
|---|---|---|
| $Mo_{12}C_{12}$ | 20.08 | -17.78 |
| | 17.08 | -17.78 |
| | 15.08 | -17.78 |
| | 14.08 | -17.78 |
| | 13.08 | -17.78 |
| | 11.08 | -17.78 |
| | 10.08 | -17.78 |
| | 09.08 | -17.79 |
| | 08.08 | -17.80 |
| | 07.08 | -18.34 |

**Figure S5.** Two orthogonal atomic structure views of $Mo_6C_4$ (a) most stable $C_{4v}$ isomer and (b) less stable (by 0.7 eV) $C_{2v}$ isomer. Color-code as in Figure S1.

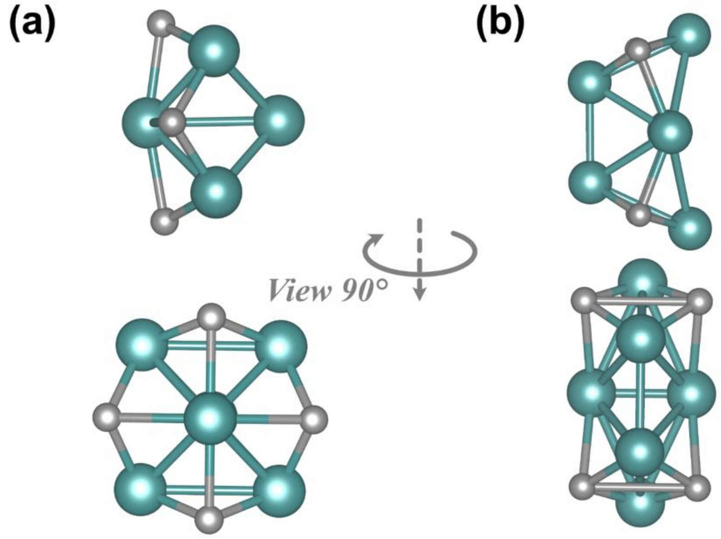

**Figure S6.** Geometric structures of the minima found for $MoC_y$ clusters adsorbed on flat graphene, with their corresponding $E_{ads}$ values, and relative energies, $\Delta E$, with respect to the most stable adsorption configuration. Color-code as in Figure S1.

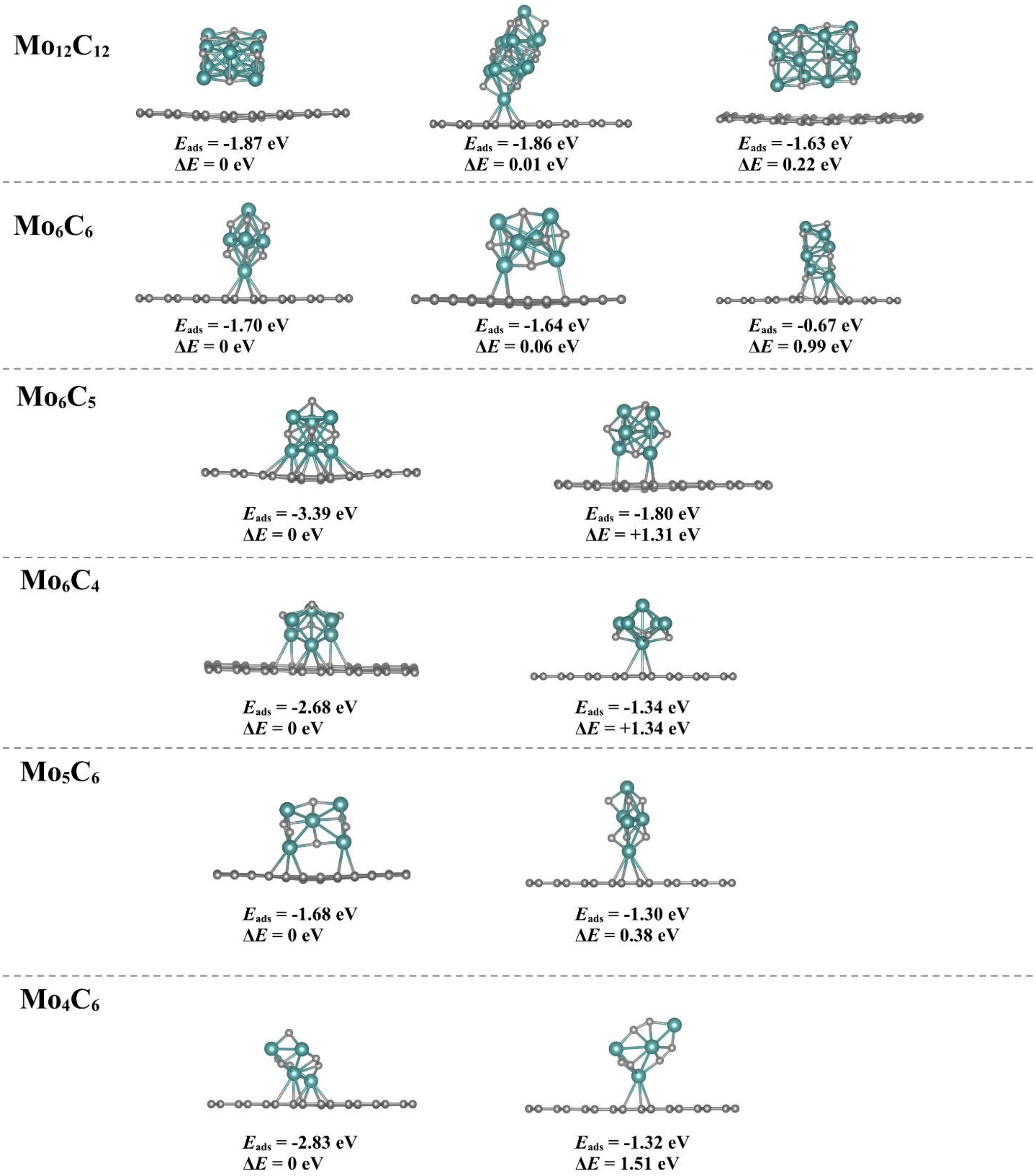

**Table S2.** Calculated adsorption energies, $E_{ads}$, deformation energies, $E_{def}$, attachment energies, $E_{att}$, and root mean square displacement (RMSD) of $MoC_y$ clusters supported on FG. All energy values are given in eV, while RMSD is given in Å.

| | $E_{ads}$ | $E_{def}^{MoC_y}$ | $E_{def}^{FG}$ | $E_{att}$ | RMSD |
|---|---|---|---|---|---|
| **$Mo_{12}C_{12}$ (Edge)** | -1.87 | 0.11 | 0.15 | -2.13 | 0.0385 |
| **$Mo_{12}C_{12}$ (Corner)** | -1.86 | 0.23 | 0.05 | -2.14 | 0.0579 |
| **$Mo_6C_6$ (Edge)** | -1.70 | 0.25 | 0.24 | -1.90 | 0.0581 |
| **$Mo_6C_6$ (Corner)** | -1.64 | 0.16 | 0.04 | -2.13 | 0.0413 |
| **$Mo_6C_5$** | -3.39 | 0.87 | 0.29 | -4.55 | 1.8035 |
| **$Mo_6C_4$** | -2.68 | 0.22 | 0.13 | -3.03 | 0.0710 |
| **$Mo_5C_6$** | -1.68 | 0.78 | 0.21 | -2.67 | 1.8294 |
| **$Mo_4C_6$** | -2.83 | 0.44 | 0.13 | -3.39 | 0.0832 |

**Figure S7.** Side views of the two strongest adsorption configurations of (a) $Mo_{12}C_{12}$ clusters and (b) $Mo_6C_6$ clusters on FG, along with CDD plots, and (c) the energetic variables, including $E_{ads}$, $E_{att}$, and $E_{def}^{MoC_y}$ and $E_{def}^{FG}$. Electron depletion/accumulation isosurfaces are depicted blue/yellow at isovalues of ±0.03 e·Å$^{-3}$. The black arrows and numbers indicate the direction and magnitude of the total charge transfer, while black lines are plane-averaged CDD. Color-code as in Figure S1.

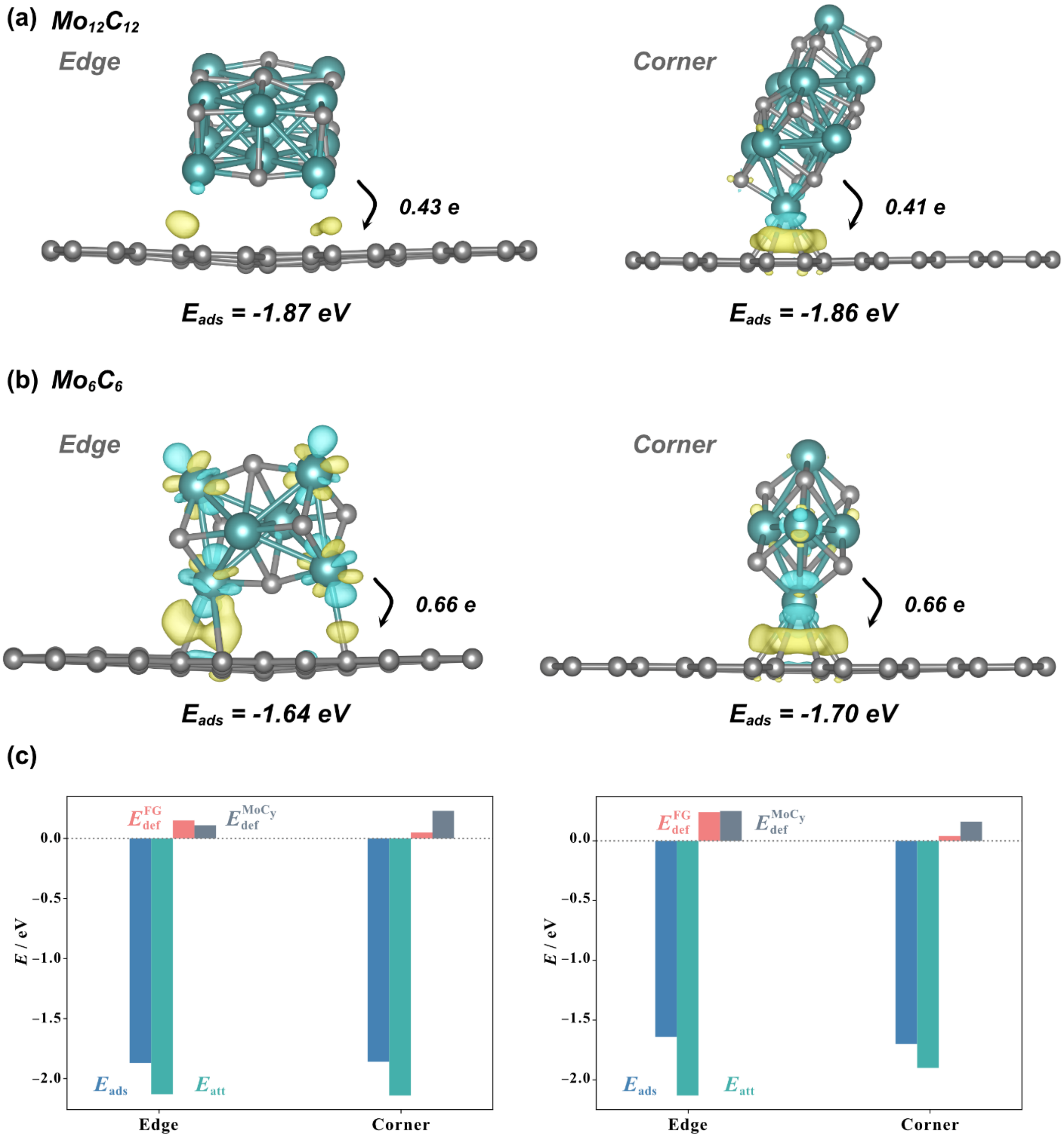

**Figure S8.** Three-view projections of the most stable adsorption configurations of MoCy clusters adsorbed on flat graphene (FG) and the corresponding CDD plots, with electron depletion/accumulation isosurfaces are depicted blue/yellow at isovalues of ±0.03 e·Å$^{-3}$. Color-code as in Figure S1.

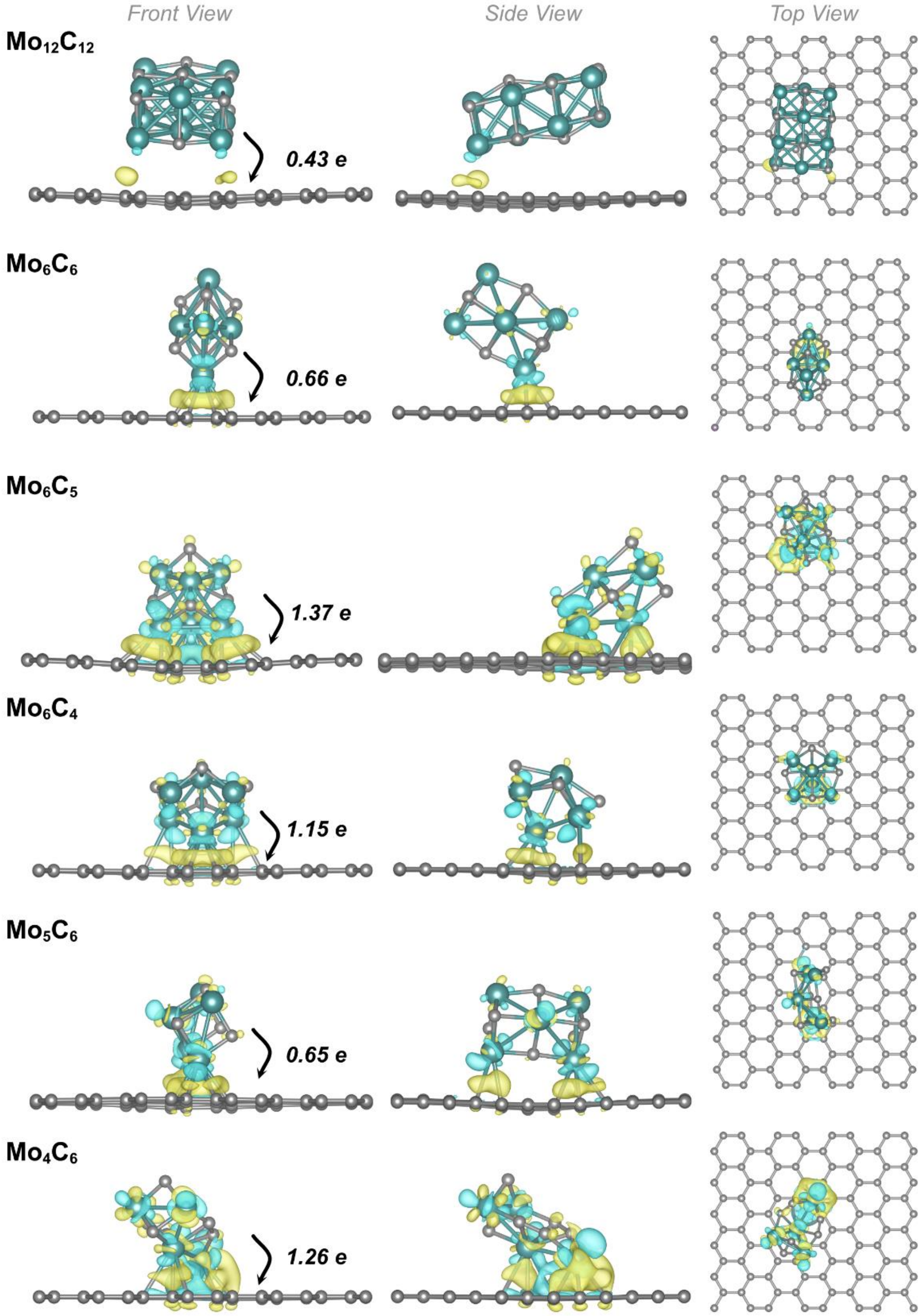

**Figure S9.** Morphology comparison of $Mo_5C_6$ cluster in (a) vacuum and (b) after adsorption on FG. Color-code as in Figure S1.

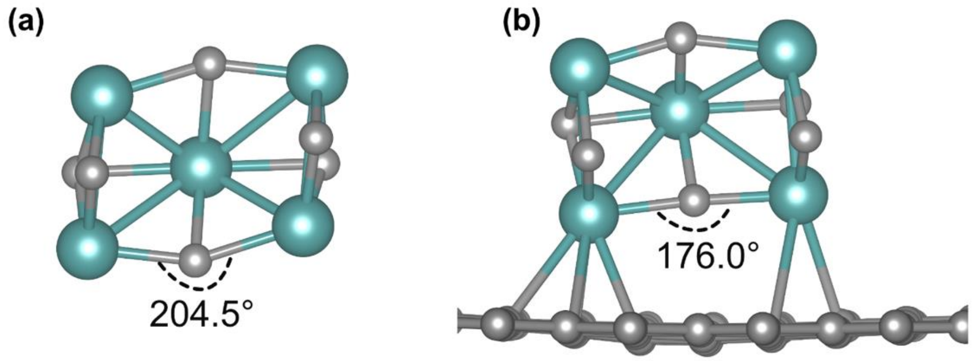

**Figure S10.** Side views of most stable adsorption configurations of $MoC_y$ clusters on CGs at different curvatures, both on the concave and convex sides, plus the corresponding CDD plots, with electron depletion/accumulation isosurfaces are depicted blue/yellow at isovalues of ±0.03 $e \cdot Å^{-3}$. Atomic color-code as in Figure S1.

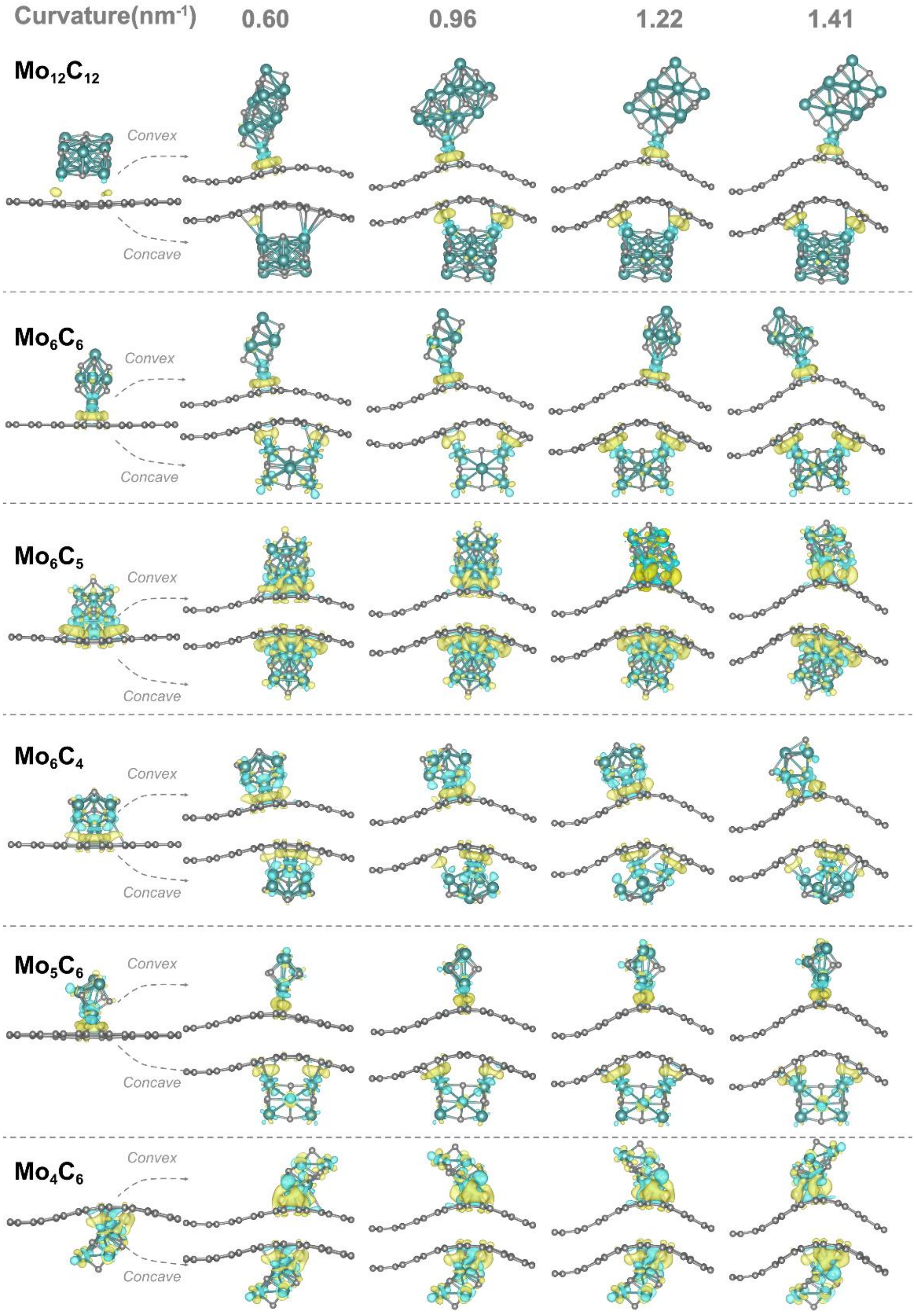

**Figure S11.** Bader charges, $Q$, on various $MoC_y$ NPs adsorbed on the concave/convex sides of modeled CGs with different curvatures, signed here with different $\kappa$ values, along with their linear regression relationships.

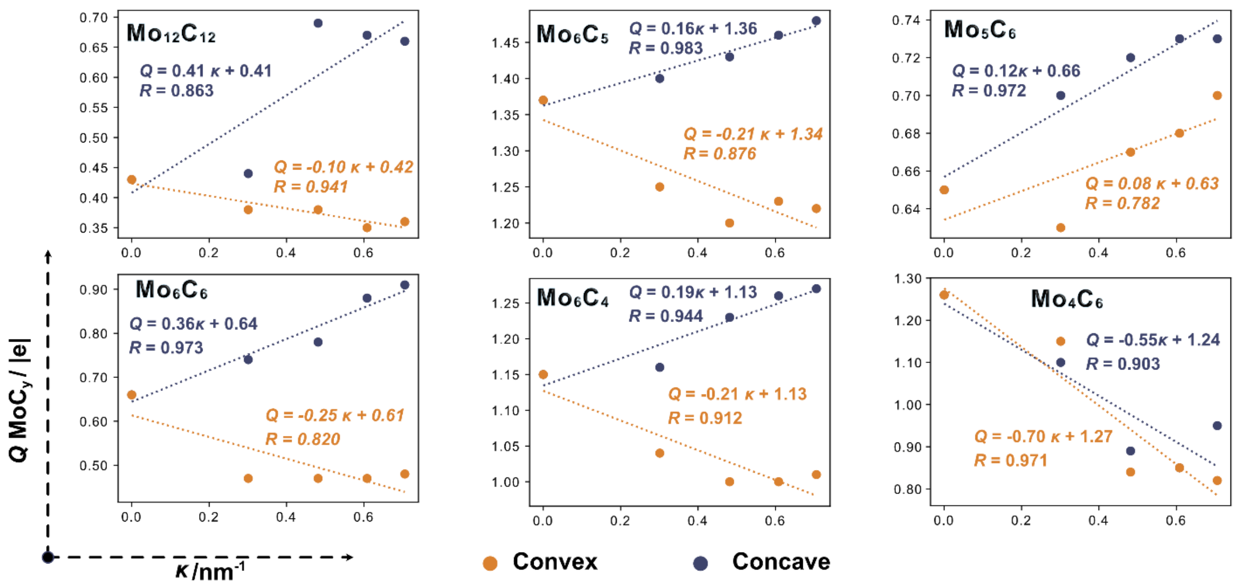

**Table S3.** Calculated adsorption, attachment, and deformation energies of $MoC_y$ clusters supported on curved graphene (CG) with different curvatures, *κ*.

| $MoC_y$ | κ / nm$^{-1}$ | $E_{ads}$ / eV | $E_{def}^{MoC_y}$ / eV | $E_{def}^{CG}$ / eV | $E_{att}$ / eV |
|---|---|---|---|---|---|
| $Mo_{12}C_{12}$ Concave | 0.60 | -2.12 | 0.10 | 0.13 | -2.34 |
| | 0.96 | -2.40 | 0.49 | 0.19 | -3.08 |
| | 1.22 | -2.57 | 0.38 | 0.15 | -3.10 |
| | 1.41 | -2.71 | 0.34 | 0.13 | -3.17 |
| $Mo_{12}C_{12}$ Convex | 0.60 | -1.74 | 0.23 | 0.04 | -2.00 |
| | 0.96 | -1.62 | 0.22 | 0.03 | -1.87 |
| | 1.22 | -1.58 | 0.19 | 0.03 | -1.81 |
| | 1.41 | -1.56 | 0.19 | 0.03 | -1.78 |
| $Mo_6C_6$ Concave | 0.60 | -2.00 | 0.30 | 0.23 | -2.53 |
| | 0.96 | -2.28 | 0.30 | 0.18 | -2.76 |
| | 1.22 | -2.56 | 0.67 | 0.16 | -3.38 |
| | 1.41 | -2.65 | 0.67 | 0.11 | -3.43 |
| $Mo_6C_6$ Convex | 0.60 | -1.59 | 0.15 | 0.03 | -1.78 |
| | 0.96 | -1.57 | 0.15 | 0.03 | -1.75 |
| | 1.22 | -1.54 | 0.15 | 0.03 | -1.71 |
| | 1.41 | -1.55 | 0.15 | 0.04 | -1.74 |
| $Mo_6C_5$ Concave | 0.60 | -3.64 | 0.87 | 0.22 | -4.74 |
| | 0.96 | -3.70 | 0.86 | 0.21 | -4.77 |
| | 1.22 | -3.65 | 0.84 | 0.24 | -4.74 |
| | 1.41 | -3.67 | 0.84 | 0.24 | -4.76 |
| $Mo_6C_5$ Convex | 0.60 | -2.92 | 0.93 | 0.19 | -4.04 |
| | 0.96 | -2.83 | 0.96 | 0.17 | -3.96 |
| | 1.22 | -2.69 | 0.50 | 0.41 | -3.60 |
| | 1.41 | -2.74 | 0.52 | 0.42 | -3.67 |
| $Mo_6C_4$ Concave | 0.60 | -2.75 | 0.22 | 0.13 | -3.11 |
| | 0.96 | -2.89 | 0.21 | 0.18 | -3.28 |
| | 1.22 | -3.06 | 0.25 | 0.15 | -3.46 |
| | 1.41 | -3.16 | 0.22 | 0.14 | -3.52 |
| $Mo_6C_4$ Convex | 0.60 | -2.52 | 0.20 | 0.13 | -2.85 |
| | 0.96 | -2.53 | 0.24 | 0.15 | -2.92 |
| | 1.22 | -2.49 | 0.19 | 0.11 | -2.78 |
| | 1.41 | -2.31 | 0.17 | 0.26 | -2.75 |
| $Mo_5C_6$ Concave | 0.60 | -1.97 | 0.74 | 0.27 | -2.99 |
| | 0.96 | -2.09 | 0.69 | 0.14 | -2.92 |
| | 1.22 | -2.25 | 0.65 | 0.10 | -2.99 |
| | 1.41 | -2.31 | 0.60 | 0.07 | -2.98 |
| $Mo_5C_6$ Convex | 0.60 | -1.57 | 0.67 | 0.16 | -2.40 |
| | 0.96 | -1.59 | 0.77 | 0.21 | -2.57 |
| | 1.22 | -1.59 | 0.79 | 0.19 | -2.57 |
| | 1.41 | -1.64 | 0.80 | 0.24 | -2.69 |
| $Mo_4C_6$ Concave | 0.60 | -2.68 | 0.30 | 0.15 | -3.14 |
| | 0.96 | -2.69 | 0.44 | 0.19 | -3.33 |
| | 1.22 | -2.62 | 0.44 | 0.23 | -3.29 |
| | 1.41 | -2.48 | 0.44 | 0.28 | -3.20 |
| $Mo_4C_6$ Convex | 0.60 | -2.72 | 0.42 | 0.12 | -3.27 |
| | 0.96 | -2.70 | 0.46 | 0.15 | -3.32 |
| | 1.22 | -2.69 | 0.41 | 0.18 | -3.29 |
| | 1.41 | -2.62 | 0.40 | 0.34 | -3.36 |

**Figure S12.** A charge density slice and heat map of $Mo_4C_6$ NP in vacuum (left), and schematic representation of the π-π repulsion between the $Mo_4C_6$ NP and graphene sheet π states, shown as a counter value of normalized relative electron density. Comparative Bader charges analysis between the single carbon atom and the carbon dimer.

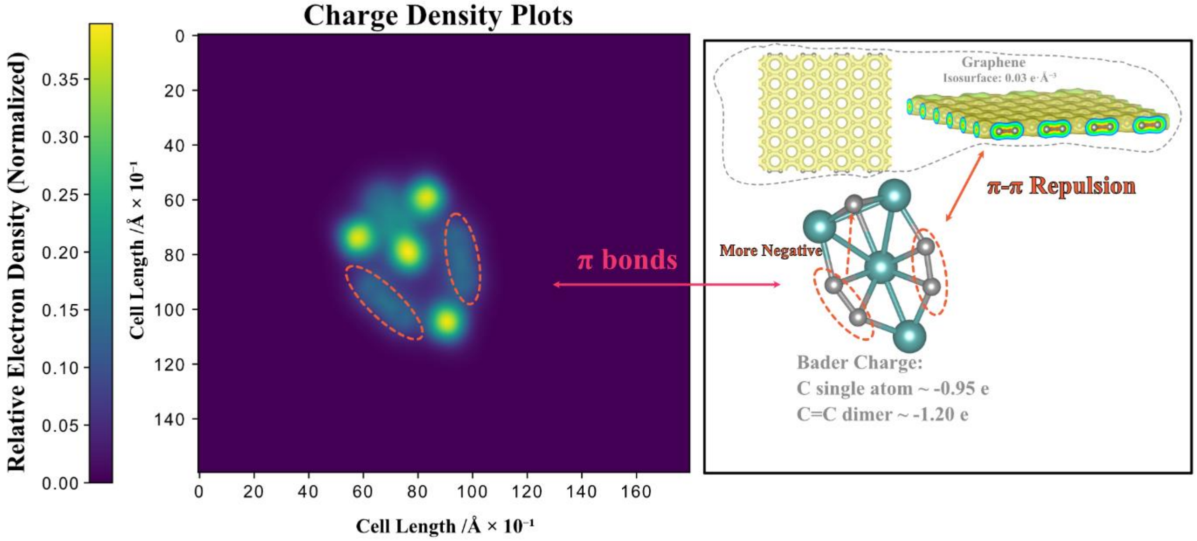

Supplement: Supplementary file 1 — am4c17904_si_001.pdf [file am4c17904_si_001.pdf]
